# Hybrid brain/neural interface and autonomous vision-guided whole-arm exoskeleton control to perform activities of daily living (ADLs)

**DOI:** 10.1186/s12984-023-01185-w

**Published:** 2023-05-06

**Authors:** José M. Catalán, Emilio Trigili, Marius Nann, Andrea Blanco-Ivorra, Clemente Lauretti, Francesca Cordella, Eugenio Ivorra, Elaine Armstrong, Simona Crea, Mariano Alcañiz, Loredana Zollo, Surjo R. Soekadar, Nicola Vitiello, Nicolás García-Aracil

**Affiliations:** 1 Robotics and Artificial Intelligence Group of the Bioengineering Institute, Miguel Hernandez University, 03202 Elche, Spain; 2 BioRobotics Institute, Scuola Superiore Sant’Anna, 56025 Pontedera, Italy; 3 Department of Excellence in Robotics & AI, Scuola Superiore Sant’Anna, Pisa, Italy; 4 Clinical Neurotechnology Laboratory, Charité, Universitätsmedizin Berlin, 10117 Belin, Germany; 5 Laboratory of Biomedical Robotics and Biomicrosystems, Università Campus Bio-Medico di Roma, 00128 Rome, Italy; 6 University Institute for Human-Centered Technology Research (Human-Tech), Universitat Politècnica de València, 46022 Valencia, Spain; 7 CEDAR Foundation, 56025 Belfast, Northern Ireland, UK; 8 IRCCS, Fondazione Don Carlo Gnocchi, Milan, Italy

**Keywords:** Assistive robotics, Brain–machine interface, User intention prediction, Multimodal system, Brain injury, Spinal-cord injury

## Abstract

**Background:**

The aging of the population and the progressive increase of life expectancy in developed countries is leading to a high incidence of age-related cerebrovascular diseases, which affect people’s motor and cognitive capabilities and might result in the loss of arm and hand functions. Such conditions have a detrimental impact on people’s quality of life. Assistive robots have been developed to help people with motor or cognitive disabilities to perform activities of daily living (ADLs) independently. Most of the robotic systems for assisting on ADLs proposed in the state of the art are mainly external manipulators and exoskeletal devices. The main objective of this study is to compare the performance of an hybrid EEG/EOG interface to perform ADLs when the user is controlling an exoskeleton rather than using an external manipulator.

**Methods:**

Ten impaired participants (5 males and 5 females, mean age 52 ± 16 years) were instructed to use both systems to perform a drinking task and a pouring task comprising multiple subtasks. For each device, two modes of operation were studied: synchronous mode (the user received a visual cue indicating the sub-tasks to be performed at each time) and asynchronous mode (the user started and finished each of the sub-tasks independently). Fluent control was assumed when the time for successful initializations ranged below 3 s and a reliable control in case it remained below 5 s. NASA-TLX questionnaire was used to evaluate the task workload. For the trials involving the use of the exoskeleton, a custom Likert-Scale questionnaire was used to evaluate the user’s experience in terms of perceived comfort, safety, and reliability.

**Results:**

All participants were able to control both systems fluently and reliably. However, results suggest better performances of the exoskeleton over the external manipulator (75% successful initializations remain below 3 s in case of the exoskeleton and bellow 5s in case of the external manipulator).

**Conclusions:**

Although the results of our study in terms of fluency and reliability of EEG control suggest better performances of the exoskeleton over the external manipulator, such results cannot be considered conclusive, due to the heterogeneity of the population under test and the relatively limited number of participants.

**Supplementary Information:**

The online version contains supplementary material available at 10.1186/s12984-023-01185-w.

## Background

The aging of the population and the progressive increase of life expectancy in developed countries is leading to a high incidence of age-related cerebrovascular diseases, which affect people’s motor and cognitive capabilities and might result in the loss of arm and hand functions [1, 2]. In addition, spinal cord or central nervous system injuries are the most common causes of long-term disabilities in adulthood [3]. Such conditions have a detrimental impact on people’s quality of life, forcing them to rely on external sources of assistance, such as family members or other caregivers, to perform common ADLs [4–6]. Assistive robots have been developed to help people with motor or cognitive disabilities to perform ADLs independently.

Numerous examples in the literature of robotic devices for the assistance of people with chronic diseases have shown good results in terms of restoring ADLs capabilities and improved autonomy and independence in these tasks [7, 8]. Whereas most of the robotic systems for ADLs assistance in the state of the art include external manipulators and exoskeletal devices have also been proposed [9]. To be effective in restoring ADLs, exoskeletons must be endowed with cognitive interfaces to gather information from the user and, ideally, from the environment, generate the appropriate assistive actions to fulfill the tasks. brain–computer interface (BCI) have the capability to decode brain-generated commands (e.g., via motor imagery) and translate them into the control of assistive devices, allowing users with complete or partial loss of movement functions to trigger or modulate robotic actions to accomplish motor tasks. Examples of applications of BCI in the control of wearable robotic devices include the use of electroencephalography (EEG) [10, 11] or magnetoencephalography (MEG) [12–15]. Multi-modal control strategies also employing EEG or MEG in addition to electrooculography (EOG) or electromyography (EMG) have been explored, to improve BCI accuracy or to increase the number of classes to recognize, thus the number of controllable degrees of freedom of the system [16, 17].

In the context of BCI-based multi-modal architectures for the control of upper-limb exoskeletons in daily-life scenarios, a few robotic platforms have been developed over the last few years. The multi-modal haptic interface in [18] exploited real-time EEG recordings to detect lateralized readiness potential (LRP) and modulate the parameters of an impedance controller for an upper-limb exoskeleton. The shared control architecture in [19] used a gaze tracking system to localize objects in a semi-structured environment, while an EEG interface exploited motor imagery to modulate speed, acceleration, and jerk of the active joint of an upper-limb exoskeleton assisting reaching tasks. In the framework of the MUNDUS project [20], neuromuscular electrical stimulation was combined with a whole-arm exoskeleton and different interfaces, including a USB button, a BCI, and a gaze tracker, to perform drinking tasks.

We have previously conducted some studies with healthy subjects [21, 22] and stroke survivors [23] to demonstrate the feasibility and safety of a shared hybrid EEG/EOG control paradigm to operate an autonomous whole-arm exoskeleton through a series of sub-tasks of daily living. The proposed hybrid EEG/EOG control scheme is advantageous for the performance of ADLs in severely impaired individuals. Indeed, the EEG interface has been widely used for the control of external assistive devices and it would allow adapting their control to patients with tetraplegia or severe functional impairment, for whom other types of interfaces (such as EMG) would be unreliable or unfeasible [24]. In this system, an EEG interface using surface electrodes exploits modulations of sensorimotor rhythm (SMR, 8–12 Hz) and SMR event-related desynchronization to trigger actions for a robotic hand exoskeleton module, whereas non-invasive EOG electrodes trigger reaching movements via a shoulder-elbow-wrist exoskeleton module. An unsolved question remains about the effectiveness and reliability of such a hybrid, non-invasive interface in restoring ADLs when used by different target populations (e.g., individuals with spinal cord or brain injuries), and with different assistive devices (e.g., exoskeletons versus external manipulators). A recent study on the actual demands of potential end users of assistive robotic devices showed that, among typical ADLs, the use of external robotic manipulators was preferred for eating or moving nearby objects, while activities such as dressing, controlling the wheelchair, or using the toilet showed a higher demand for the employment of upper-limb exoskeletons [25]. Nevertheless, providing an intuitive, reliable, and safe control of assistive robotic devices is still an open challenge.

The main objective of this study is to compare the performance of the hybrid EEG/EOG interface to perform ADLs when the user is controlling an exoskeleton rather than using an external manipulator (Jaco®, Kinova, Canada). In particular, we hypothesize that contingent proprioceptive feedback induced by EEG modulation of robot-assisted movements could improve BCI performances, as suggested in other studies [26–28]. The EEG modulation was applied to the control of the opening and closing actions of the hand exoskeleton rather than the shoulder-elbow wrist exoskeleton, to cope with the limitations of bandwidth and reliability when controlling multiple degrees of freedom (DOF) [22]. For each device, two modes of operation were studied: synchronous mode (the user received a visual cue indicating the sub-tasks to be performed at each time) and asynchronous mode (the user started and finished each of the sub-tasks independently). Notably, in both cases, the execution of the sub-tasks was supervised by a finite-state machine, which forbade concurrent triggering of reaching/retracting and hand opening/closing movements. After each condition, participants were asked to fill out the NASA-TLX questionnaire to evaluate the task workload [29]. For the trials involving the use of the exoskeleton, a custom Likert-Scale questionnaire was used to evaluate the user’s experience in terms of perceived comfort, safety, and reliability [30].

## Methods

### Participants

The participant’s group includes 10 users whose demographics, impairment type, and Barthel Index [31] are listed in Table 1. Before entering the study, all participants provided written informed consent. Inclusion criteria were: (i) neurological condition such as spinal cord injury, acquired brain injury, stroke, multiple sclerosis, motor neuron disease, and muscular dystrophy; (ii) those who benefit from the technology; (iii) living within Northern Ireland; (iv) age above 18 years old. Exclusion criteria were: (i) people who were receiving acute services or other rehabilitation of support services, (ii) those whose participation would have been detrimental to their wellbeing, (iii) those who withheld consent or were unable to give consent (verbal or written), (iv) history of epilepsy, (v) photosensitive people, (vi) people living with any secondary conditions such as mental illness.

The study protocol complied with the Research Ethics Framework of the Economic and Social Research Council (ESRC 2010). Moreover, we liaised with Office Research Ethics Northern Ireland (ORECNI), which is the regional affiliate of the United Kingdom National Research Ethics Service, to perform a review that ensured establishing ethical methods free from bias and undue influence.

It should be noted that in the study, patient 10 decided to abandon the experiment due to fatigue, so she could not finish the experiment. In addition, due to technical problems, the asynchronous mode session with the external robotic manipulator of patient 1 was lost. These limitations have been considered when analyzing the data presented in this study.

**Table 1:** Characteristics of the patients together with the Barthel Index [30, 31]

Patients	Sex	Age	Diagnostic	Laterality	Barthel Index
1	M	66	Encephalitis	Right	20/20
2	F	62	Stroke	Right	18/20
3	M	52	Traumatic brain injury, stroke	Right	20/20
4	F	20	Spinal cord injury C6/7	Right	10/20
5	F	N/A	Traumatic brain injury-lived with locked in	Right	16/20
6	M	55	Stroke	Right	16/20
7	F	N/A	Cerebral palsy. ataxia	Right	19/20
8	M	N/A	Traumatic brain injury	Right	19/20
9	M	N/A	Spinal cord injury	Right	16/20
10	F	56	Stroke	Left	13/20

Some information has been omitted voluntarily by the participants

*N/A* not available value

### Whole-arm exoskeleton

**Fig. 1:**
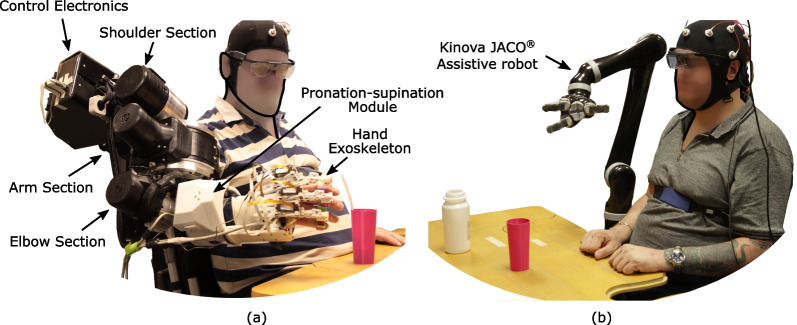
Robotic devices. **a** Illustration of the different components of the whole-arm exoskeleton compared to **b** Kinova Jaco® assistive robot-based system

The whole-arm exoskeleton comprises three components (Fig. 1): the shoulder-elbow exoskeleton NeuroExos Shoulder-elbow Module $\beta$ (NESM-$\beta$ [32, 33], a pronation-supination module [34] and a hand exoskeleton [35–37].

### Shoulder–elbow exoskeleton (NESM-$\beta$)

The NESM-$\beta$ [32, 33] is a shoulder–elbow exoskeleton equipped with four series–elastic actuators. Active joints feature the shoulder adduction–abduction (sAA), shoulder flexion–extension (sFE), shoulder internal–external rotation (sIEr) and elbow flexion–extension (eFE). Being the zero-configuration with the arm laying parallel to the trunk, the four active joints present the following ROMs: [$-2^{\circ}$  $+83^{\circ}$] sAA and sFE, [$-90^{\circ}$  $30^{\circ}$] sIEr and [$10^{\circ}$  $100^{\circ}$] eFE. The total weight of the arm is around 16 kg. The exoskeleton has been designed to be used either for the left or the right arm, with a mechanism to quickly flip the actuation units from one configuration to the other. In addition, passive degrees of freedom are embedded in the support structure of the exoskeleton to follow the natural movements of the shoulder elevation–depression and protraction-retraction, as well as scapula medial–lateral translation. Size regulations are included to adjust the shoulder center of rotation along the medial–lateral and anterior–posterior direction and to adjust the position of the elbow axis according to the user’s humerus length.

The NESM-$\beta$ has been designed to be integrated into an electric wheelchair, to address portability requirements and provide a more realistic environment for daily-life assistance. A support structure is placed on the rear side of the wheelchair and consists of a metallic box, hosting the exoskeleton weight relief system, and a series of adjustable tubular linkages, connected to the wheelchair by means of mechanical clutches.

The control system of the NESM-$\beta$ is organized in a hierarchical architecture with two layers. At the low-level layer, a position controller and a torque controller for each joint have been implemented, realizing respectively the so-called robot-in-charge and patient-in-charge programs. Both controllers are designed as proportional-integrative-derivative (PID) closed-loop regulators, operating on the difference between the desired variable (i.e., position or torque) and the measured one. The control electronics includes a sbRIO-9651 System On Module (National Instruments, US), endowed with a dual-core 667 MHz real-time processor, where the high-level control running at 100 Hz is implemented, and a reconfigurable fieldprogrammable- gate-array (FPGA) for the low-level control layer, running at 1 kHz.

### Pronation–supination module

The pronation–supination module, an evolution of the version presented in [34], is a 1-active DOF device designed for the assistance of the pronation/supination movement that can be used on both right and left arms. It allows a total range of motion (ROM) of $152^\circ$, which is sufficient to perform most activities of daily living [34]. The module includes a Maxon brushless EC motor/gear combination that can provide an output torque of 0.45 Nm. For the transmission of the movement a circular toothed guide placed on the outside of the forearm has been used, which provides a reduction to the mechanism of 8:1, obtaining in the assisted joint a torque of 3.61 Nm. The Maxon EPOS2 24/2 Positioning Controllers have been used.

The mechanical design was improved with respect to the previous prototype [34], resulting in a smaller and lighter device. In addition, the physical human–robot interfaces have been produced in different sizes, to increase the adaptability to users with different anthropometries. Finally, a passive regulation was included for the placement of the hand exoskeleton to adjust the forearm cuff’s attachment in a range between 23.5 to 30.5 cm from the elbow joint. The total weight of the device is around 1.17 kg.

### Hand exoskeleton

The hand exoskeleton [35–37] is an under-actuated robotic device aimed to assist the user during the grasping of objects. In particular, it has been designed to grasp cylindrical-shaped objects such as cups, bottles, or adapted cutlery. The hand exoskeleton has four active degrees of freedom corresponding to index finger flexion–extension, middle finger flexion–extension, ring and little fingers flexion–extension, and thumb flexion–extension in opposition. The three degrees of freedom corresponding to the long fingers are driven by three equal finger modules, transferring a flexion/extension motion to the phalanxes by means of a bar linkage commanded by a linear actuator. As for the thumb module, a design choice was not to apply a constrained motion directly to the thumb joints, to comply with users having non-physiological thumb postures due to muscle hypertonia or similar conditions. Therefore, the thumb module consists of physical human–robot interface with a wide interface area that offers comfortable support to the user’s thumb, and a lever mechanism to achieve opposition without requiring precise control of the individual joints. All modules have a position feedback signal, that allows the low-level controller to perform a PID position control of the user’s finger pose.

### External manipulator

Jaco® robot produced by Kinova (Boisbriand, Canada) [38] was used as external manipulator (Fig. 1). The robotic arm consists of a fixed base linked to six carbon fiber shells and a gripper through rotation actuators. It has been designed to be installed on a motorized wheelchair and used by persons living with upper-extremity mobility limitations. Users can control it to reach, move and manipulate objects in their surroundings, with the benefit of enhanced autonomy in daily-living activities.

The Jaco® robot can be easily re-located and fixed on one side of the wheelchair (right or left). The control system of the robot, fully implemented in the robot operating system (ROS) environment, is implemented in a two-layer architecture. The first layer is deputed to the low-level control of the robot. This is responsible for controlling the robot actuators to perform the desired movements safely and accurately. On the higher level, a communication layer is implemented, which receives information and commands from external inputs and sends commands to the low-level layer to control the robot accordingly. At the same time, it is used to send externally information about the current status of the robot (e.g., movement/rest states).

### EEG/EOG control interface

To record EEG/EOG, a 5-channel, wireless EEG (LiveAmp®, Brain Products GmbH, Gilching, Germany) was recorded from the following conventional 10/20 system recording sites: F3, T3, C3, Cz and P3 using polyamide-based solid-gel electrodes. Ground and reference electrodes were placed at AFz and FCz. Two additional channels were used to detect horizontal eye movements (HOV) using EOG signals recorded from the left and right outer canthus. EEG and EOG were sampled at 1 kHz and band pass-filtered at 0.1–30 Hz. To increase signal-to-noise ratio, EEG was pre-processed using a surface Laplacian filter [39]. A customized version of the open-source BCI2000 software was used to translate the EEG and EOG signals into whole-arm exoskeleton control commands. Sensorimotor rhythm-event-related desynchronization (SMR-ERD) were calculated using the power method by [40]. The system was calibrated at the beginning of the experimental session and kept unchanged throughout the session. Detection thresholds for SMR-ERD and HOV were identified and set as in [41]. For calibration of the EEG/EOG control interface, a reference value (RV) of SMR-ERD related to externally-paced imagined hand opening or closing movements of the right hand was calculated by using a power spectrum estimation based on an autoregressive model of order 100 (Burg algorithm). Calculation of the RV comprised a total of 42 trials, each lasting 5 s, followed by an inter-trial interval (ITI) of 4 s, during which participants were inactive (rest condition). For online classification of SMR-ERD, a frequency filter with an frequencies of interest (FOI) of ±1.5 Hz was used. A detection threshold for movement initiation and execution was calculated based on the additional 42 trials, during which participants received online visual feedback of SMR-ERD provided on the display in front of them. The detection threshold was set to the average of elicited SMR-ERD across all trials and used for online EEG control. SMR-ERD was translated into a control command if detection threshold was exceeded. As a next step, participants were instructed to perform 10 externally paced horizontal eye movements to the left (HOVl) or HOV to the right (HOVr) following a visual cue while band pass-filtered EOG (0.1–5 Hz) was recorded. HOV detection threshold was at 70% of the average EOG signal recorded during maximum HOV.

### Computer vision system

Most ADLs require the capability to perform reaching and manipulation tasks precisely, interacting with external objects in a complex unstructured environment. To provide robotic assistance in such environment, real-time object tracking should be performed, dealing with the possible disturbances arising from interaction with the objects. The solution should be efficient, accurate, scalable, and robust to changes in the environment (i.e., occlusions, uncontrolled variations in lighting).

Several methods have been proposed to tackle this problem. However, despite the great advances in the field, especially using deep learning techniques, among the most popular methods are the SSD-6D [42], BB8 [43], Pose-CNN [44], and [45, 46] methods, real-time object classification and tracking is challenging when dealing with non-textured objects. Some authors have used commercial tracking systems like Optitrack or ART Track [47–49]. The main limitation of these devices is the necessity to modify the objects to track through the inclusion of optical markers, to reconstruct their position and orientation.

For well-textured objects, several methods based on appearance descriptors like SURF or SIFT [50] can be employed. However, the most common objects in our daily living are textureless.

**Fig. 2:**
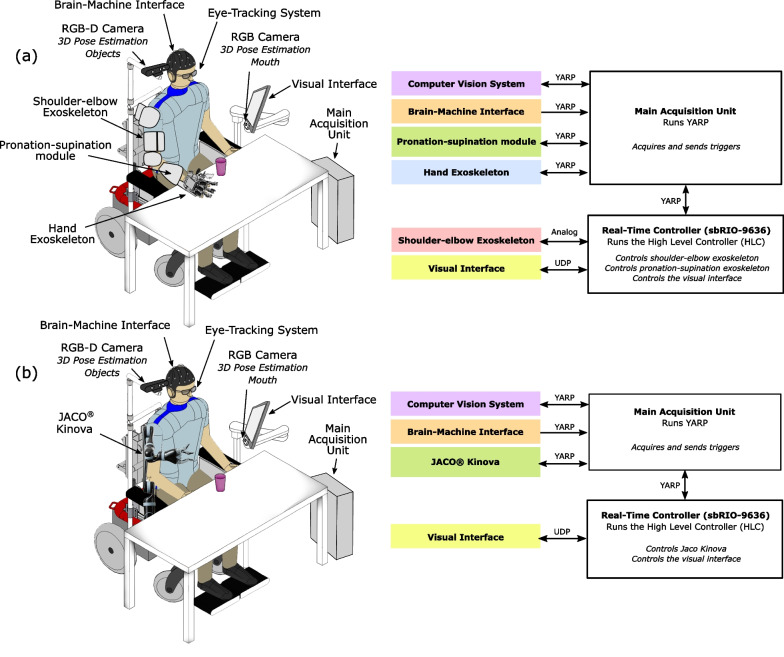
Communication architecture diagrams. Overview of components and communication architecture based on YARP, UDP, and analog communication for **a** the whole-arm exoskeleton-based system compared to **b** Kinova Jaco® assistive robot-based system

The proposed computer vision system is based on the use of three devices (Fig. 2). The first one is Tobii Pro Glasses 2 (Tobii Pro AB, Sweden). This eye-tracking system allows the user to select the desired object (Eye-Tracking Detection method employed has been evaluated and presented in detail in [51]). The second one is the Orbbec Astra S RGB-D camera (Orbbec, Michigan, USA) used for the 3D pose estimation of textureless objects. This camera is placed one side over the head of the user pointing to the workspace of the robot. And finally, a generic Full HD 1080p camera (ELP Web Cam Full HD 1080p 30fps) was placed below the user interface screen pointing to his/her face to implement the 3D pose estimation of the mouth. This computer vision system was tested in real conditions with patients and was also thoroughly evaluated both qualitatively and quantitatively [51].

The 3D poses estimated by the computer vision system define the target positions of the elements with which it is desired to interact with the robotic device. The trajectory executed by each robotic device is established according to this target position. In the case of the Jaco® robot, the Kinova’s trajectory planner is used. In contrast, the exoskeleton trajectory planning system is based on an algorithm that combines Learning by Demonstration with the computation of Dynamic Motion Primitives and machine learning techniques [52].

### System component communication

The communication among all the system’s module was managed by the messaging system Yet Another Robotic Platform (YARP) [53, 54]. YARP external nodes were created in Windows environment in C++ and LabVIEW and in Linux environment in C++ in the case of Jaco Robot for sending and receiving data to and from other nodes. Each node receiving and sending data was identified by a label and had a unique communication port on the YARP server. For the transmission and reception of packages, a frequency of 20 Hz was established. The YARP server was designed in such a way that all nodes could connect independently, so in case of unexpected disconnection of a node, the others could continue sending and receiving data without jeopardizing communication. The modularity of YARP allows simply creating or substituting a node in case a new hardware module is included into the system, without affecting communication among the other modules. The scheme of the communication architecture for both exoskeleton and external manipulator platforms is shown in Fig. 2.

### Experimental setup and protocol

In this study, two different experimental setups were used, differing only from the use of a whole arm exoskeleton or an external robotic manipulator-based system (Fig. 2). On the one hand, the participants will use a whole-arm exoskeleton-based system, while on the other hand an external robotic manipulator-based system. The only difference between these two setups is the robotic device. Both systems consisted of a motorized wheelchair endowed with the cameras for 3D pose estimation and computer screen running a visual interface. According to the experimental setup, either the whole-arm exoskeleton or the manipulator was fixed on the wheelchair prior to the experiments.

**Fig. 3:**
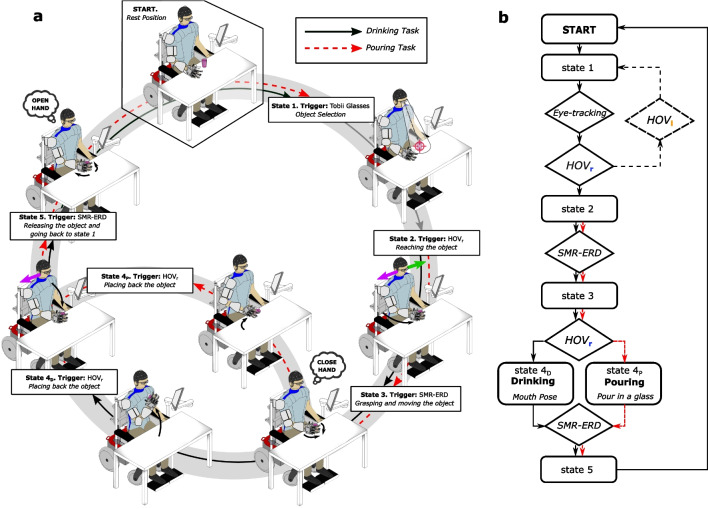
Shared-human robot control strategy on a FSM triggered by the electroencephalography and electrooculography interface (EEG/EOG) and the eye-tracking system for the drinking task (black arrow) and the pouring task (red dashed arrow). **a** Visualization of the whole-arm exoskeleton controlled by EEG/EOG and the eye-tracking system (the same with the Jaco®). Purple arrows indicate horizontal oculoversions EOG to the right (HOVr) and green arrow to the left (HOVl), while “close hand” and “open hand” indicate EEG desynchronization of sensorimotor rhythms (SMR-ERD, 9–15 Hz) related to motor imagery of grasping and releasing motions. Black arrows represent the actions of the whole-arm exoskeleton. **b** Flowchart of the whole-arm exoskeleton control loop for drinking and pouring (the same with the Jaco®)

Upon arrival, participants were asked to move to the wheelchair and were equipped with the wearable devices, including the gaze tracker, the BCI and, where applicable, the whole-arm exoskeleton. Then, the wheelchair was placed in front of a desk with two objects (a glass and a bottle). Participants familiarized with the system and received precise instructions about the actions to be performed to control a drinking task with the glass and a pouring task with the bottle and glass. Both tasks were composed of the same number of sub-tasks: (1) selection of the object (glass for drinking, bottle for pouring); (2) reaching the object; (3) grasping and drinking from the glass/pouring from the bottle; (4) placing back the object; (5) releasing the object. Sub-task 1 was initialized by the gaze tracker, sub-tasks 2 and 4 by EOG signals, whereas sub-tasks 3 and 5 were initialized by EEG signals (Fig. 3)). Participants successfully performed the drinking and pouring tasks ten times each.

Both tasks were performed in two different conditions, namely synchronous and asynchronous mode. In the first case, subjects received visual and audio feedback from a screen, to guide them in the activation of the interfaces. Given instructions were “Select” for sub-task 1, “Look right” for sub-tasks 2 and 4, and “Close (open) hand” for sub-tasks 3 and 5. A bar shows the percentage of completion of the hand opening/closing motion. Conversely, in the asynchronous mode, no instructions were provided during the task execution: a sound was generated only after the interface was correctly activated. Furthermore, in sub-task 1, audio feedback was given to warn the user about the object that was selected, to allow a new selection via the veto action if required. Users performed trials in synchronous mode first, to gain familiarity with the system, followed by trials in asynchronous mode.

The same protocol was performed both using the exoskeleton and the external manipulator. To avoid fatigue effects, trials with different hardware modules were performed on two different days. Users performed trials with the external manipulator on the first day. Subjects who could not perform the tests on separate days performed the whole protocol on the same day, with a few hours rest before starting the trials with the whole-arm exoskeleton. In this case, a new calibration procedure for the BCI was executed before starting with the experimental trials.

At the end of each session, participants were asked to fill in NASA-TLX questionnaire to estimate the workload when performing each of the tasks. Finally, at the end of the experimental session, participants were asked to fill out the Likert scale questionnaire to explore their perceptions regarding the exoskeleton. The version of the Likert scale questionnaire used in this study had the following questions:

1. I experienced side-effects or discomforts during the session.
2. I felt comfortable with the exoskeleton.
3. Control of exoskeleton was reliable and practical.
4. After completing the training, I felt safe using the exoskeleton.
5. The preparation/attaching process was comfortable for me.
6. The calibration instructions were easy to follow.
7. I felt discomfort/pain when the electrodes/exoskeleton were attached.
8. At any time, I was able to interrupt/veto the exoskeleton, when I disagreed with the control or experienced discomforts.

### Calibration procedure of the RGB-D camera

All the 3D poses of the objects estimated by the computer vision system refer to the frame of the cameras. To achieve grasping via the robotic devices, a calibration procedure between the two systems is required, similar to the hand-eye calibration problem [55].

To perform the calibration, an ArUco [56] marker is located in a known position of the robot end-effector and tracked by the cameras. Then, via forward kinematics of the robot the position and orientation of the reference system of the robot in the reference system of the cameras can be expressed. However, there are some inaccuracies in the estimated 3D pose of the ArUco marker, not only due to variations in the scenery (light intensity, obstacles, etc.) but above all due to aberrations of the camera. This has been mitigated by the calibration of the intrinsic and extrinsic parameters of the camera, but it has not been enough to ensure a high-security level for users. To solve this problem, an optimization algorithm has been employed to increase the accuracy of the calibration. This optimization algorithm implements a shape registration method to estimate the transformation between a 3D point cloud expressed in the robot base (forward kinematics of the robot) and the same 3D point cloud expressed in the camera system (ArUco marker). The method has been presented and evaluated in [51].

### Shared human–robot control

The shared human–robot control system was based on a finite-state machine (FSM) similar to the one presented in [21]. The FSM was running on the high-level control layer of the NESM-$\beta$ exoskeleton at 100 Hz and implemented the following states (Fig. 3):

- *State 1*. Object selection: selection of the object is performed by means of the gaze tracker. An ID associated to the object automatically activates the execution of the drinking task (glass detected) or the pouring task (bottle detected). A veto action, activated by the detection of a left HOV, is implemented to abort the current object selection and activate a different task.
- *State 2*. Reaching the object: in this state, the detection of HOV triggers the arm exoskeleton/external manipulator to perform the reaching movement toward the object, guided by the motion capture system.
- *State 3*. Grasping and moving the object: As long as the SMR-ERD signal is below the detection threshold, the closing motion of the hand exoskeleton is commanded, until completion. Hand motion is stopped if the SMR-ERD signal rises above the calibration threshold. Once the hand is fully closed, the arm exoskeleton/external manipulator automatically moves toward the mouth (drinking task) or toward the glass to be filled (pouring task) without requiring any additional action from the user.
- *State 4*. Placing back the object. In this state, the detection of HOV triggers the arm exoskeleton/external manipulator to move back to the position where the object was grasped.
- *State 5*. Releasing the object and going back to state 1. Similarly, to state 3, the SMR-ERD signal is used to control the opening motion of the hand exoskeleton to release the object. Once the hand is fully opened, the arm exoskeleton/external manipulator automatically moves back to the initial position (State 1).

### Data collection and analyses

The time to open/close the hand exoskeleton or the gripper of the robotic external manipulator ($t_{open/close}$) was evaluated as the time elapsed between the beginning and the end of the opening/closing movement ($t_{elapsed}$) normalized to the ERD duration ($ERD_{duration}$) (Eq. 1). In this study the ERD duration is constant as it corresponds to the opening/closing time of the hand exoskeleton mechanism (1.5 s) or the gripper mechanism of the external robotic manipulator (1.2 s). A value of 0% indicates that the time to open/close is equal to the ERD duration. If this value is greater than zero, it indicates how much this time exceeds the ERD duration.

1 $$\begin{aligned} t_{open/close}= \frac{t_{elapsed} - ERD_{duration}}{ERD_{duration}} \end{aligned}$$

EEG/EOG time to initialize (TTI) were evaluated as time since the action is visually indicated to the user until the EEG/EOG signals exceed the corresponding detection thresholds (reaction time). Nevertheless, if the EEG/EOG signal is already active before the visual indication, a negative value is computed, namely Pre-TTI, as the time between the presenting of the visual indication and the last previous activation of the EEG/EOG signal. To study the feasibility of the system, the reaction of the users was evaluated taking into account both parameters (all trials). The resulting reaction time is called TTI+Pre-TTI.

Reliability of control was defined as the time for successful initializations of at least 75% of sub-tasks. Fluent control was assumed when the time for successful initializations ranged below 3s and a reliable control in case it remained below 5 s [21].

For statistical analysis, a normality test was performed using the Shapiro-Wilk test. The results show no evidence that the parameters are normally distributed. It was therefore decided to use the Friedman test to study differences between conditions. In the post-hoc analysis, the Wilcoxon signed-rank test with the zero method proposed by Pratt [57] was used to study pairwise comparisons. Holm-Bonferroni method was used to control the family-wise error rate.

## Results

### Feasibility

**Fig. 4:**
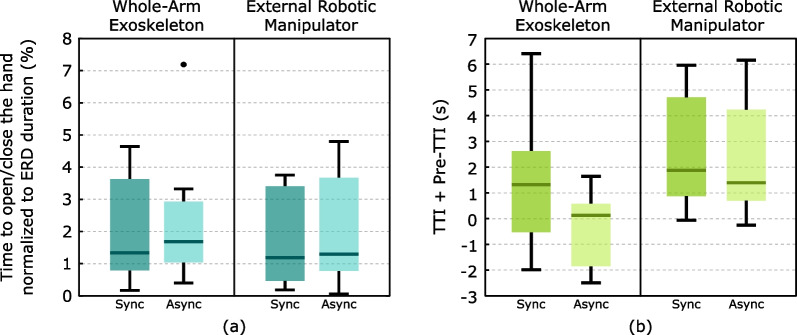
Representation of the parameters related to feasibility. **a** Time to open/close the hand normalized to ERD duration. **b** Results of the TTI+Pre-TTI parameter

Results of the time spent to fully open/close the hand exoskeleton and the gripper of the robotic external manipulator through the EEG interface normalized to ERD duration (Fig. 4a) suggest that there are no differences between conditions.

The median and interquartile range ([$q_{25}$,$q_{75}$]) of the TTI+Pre-TTI values in the case of the whole-arm exoskeleton ranged at 1.32 s [− 0.53, 2.62] s in the synchronous mode and 0.13 s [− 1.85, 0.57] s in the asynchronous mode (Fig. 4a). In both cases, 75% of the values across control modalities ranged below 3 seconds documenting reliable whole-arm exoskeleton control. On the other hand, the median and interquartile range ([$q_{25}$,$q_{75}$]) of the TTI+Pre-TTI values obtained in the case of the external robotic manipulator are slightly higher, i.e., 1.87 s [0.88, 4.71] s in the synchronous mode and 1.39 s [0.70, 4.24] s in the asynchronous mode (Fig. 4b). Nevertheless, also in this condition, 75% of the values remained below 5 seconds, documenting reliable control of the external manipulator via the BCI. Fluent control was verified for both the devices and both control modalities, with a median TTI+Pre-TTI below 3 s in all cases.

**Fig. 5:**
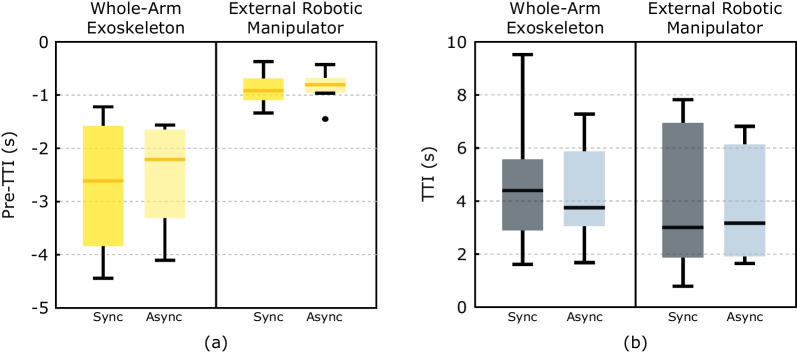
Graphical representation of the results of the Pre-TTI (**a**) and TTI-only (**b**) parameters

Fig. 5 shows the results of TTI and Pre-TTI separately. In the case of the Pre-TTI results (Fig. 5a), no statistically significant differences are observed across control modalities. However, anticipation times are greater in the case of the whole-arm exoskeleton. For this, median ([$q_{25}$,$q_{75}$]) Pre-TTI values were − 2.61 s [− 3.84, − 1.58] s and − 2.21 s [− 3.31, − 1.65] s for the synchronous and asynchronous mode respectively, whereas for the external manipulator they were respectively − 0.92 s [− 1.09, − 0.67] s and − 0.81 s [− 0.96, − 0.68] s.

When observing only the trials in which no pre-activations occurred (i.e., TTI only parameter), no statistically significant differences were found between control modalities (Fig. 2b). However, the results indicate that, in the case of the use of the exoskeleton, 75% of the TTI values remain below 6 seconds, while for the control of the external robotic manipulator it is a bit higher since 75% of the values are below 7 s. Nevertheless, when only TTI is considered, fluent control of the exoskeleton was not verified, being the median value of the TTI over 3 s in both control modalities.

### Workload

**Fig. 6:**
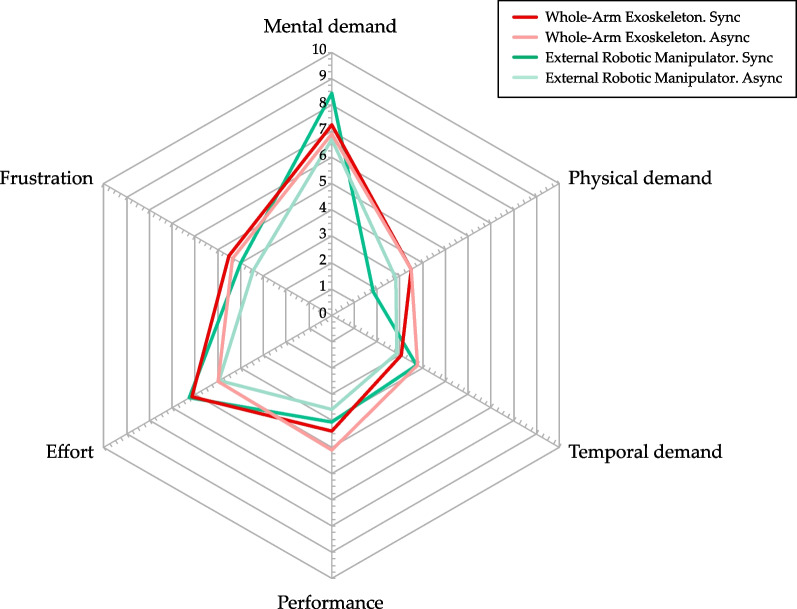
Graphical representation of the NASA TLX results

Table 2 collects the results of the NASA-TLX questionnaire. In Fig. 6, a graphical representation of the results is shown. Users report a higher Physical Demand for exoskeleton control with respect to the external robotic manipulator in both control modes.

**Table 2:** Mean and standard deviation for the NASA TLX questionnaire results for every condition

Device	Control model	Mental demand	Physical demand	Temporal demand	Performance	Effort	Frustration
External robotic manipulator	Sync	8.44	1.81	3.75	4.06	6.25	4.00
		(3.06)	(1.95)	(2.89)	(3.28)	(3.07)	(2.50)
	Async	6.69	2.81	2.88	3.56	4.94	3.44
		(1.75)	(2.71)	(2.30)	(2.62)	(2.27)	(3.01)
Whole-arm exoskeleton	Sync	7.25	3.50	3.05	4.40	6.15	4.50
		(1.25)	(2.66)	(2.92)	(2.95)	(2.70)	(3.20)
	Async	6.89	3.50	3.78	5.11	5.00	4.33
		(1.51)	(2.69)	(3.45)	(3.42)	(2.58)	(3.13)

Results also indicate that the synchronous control mode, regardless of the used device, requires more effort. After becoming familiar with the system, users reported that the asynchronous control mode would take less effort because they felt they have more control of the system and more freedom in decision-making.

Mental demand scores were in favor of the exoskeleton control, although lower performance was perceived by the subjects in the asynchronous mode. Temporal demand was comparable between exoskeleton and manipulator control, although the score for the synchronous control mode was higher (with respect to asynchronous modality) for the external manipulator, and vice versa for exoskeleton control. Finally, Frustration and Physical demand were higher for exoskeleton control in both control modes.

### Safety

**Fig. 7:**
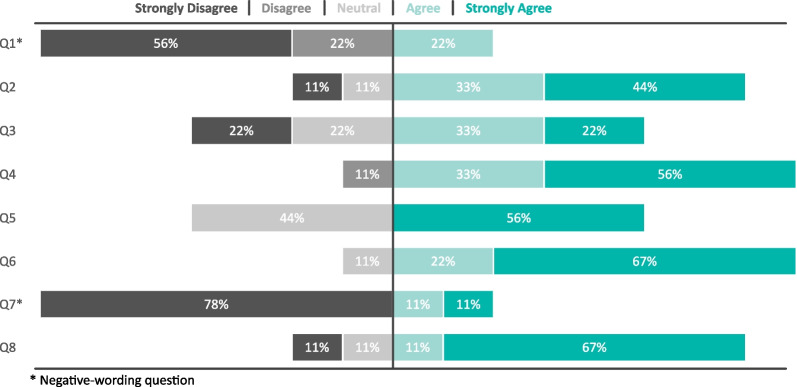
Diverging bar charts of user responses to the Likert Scale Questionnaire of five points. Questions were the following: $Q_1$: I experienced side-effects or discomforts during the session; $Q_2$: I felt comfortable with the exoskeleton; $Q_3$: Control of the exoskeleton was reliable and practical; $Q_4$: After completing the training, I felt safe using the exoskeleton; $Q_5$: The preparation/attaching process was comfortable for me; $Q_6$: The calibration instructions were easy to follow; $Q_7$: I felt discomfort/pain when the electrodes/exoskeleton were attached; $Q_8$: At any time, I was able to interrupt/veto the exoskeleton when I disagreed with the control or experienced discomforts

Fig. 7 shows diverging bar charts of users’ responses to the Likert Scale Questionnaire of five points to evaluate users’ experience with the control of the whole-arm exoskeleton. Results indicate that about 75% of users think that the system is comfortable, safe, and easy to use. Exoskeleton control was perceived as sufficiently reliable and practical by 55% of the users. 22% of the participants have a neutral opinion, while the other 22% think it is not.

At the end of the session, none of the participants reported any discomforts or side effects neither during whole-arm exoskeleton control nor the external manipulator robotic control.

## Discussion

Brain-machine interfaces combined with wearable robotic devices have been successfully demonstrated to restore movement functions in individuals with brain lesions or neuropathies [41]. In this study, we compared the control of a whole-arm exoskeleton and an external manipulator by means of a hybrid EEG/EOG interface in individuals with brain or spinal cord injuries. For the purpose of restoring ADLs capabilities, the two systems offer different and complementary advantages. Whereas the external manipulator is expected to be intrinsically safer and easier to operate due to the minimal physical human-robot interaction, a robotic exoskeleton could enable a more active user’s involvement in the task, at the cost of higher complexity from a mechanical and control view perspective. Evidence suggests that BCI-controlled exoskeleton could have beneficial effects also from a neurorehabilitation perspective, promoting neuroplasticity and functional recovery [11, 13]. In people with spinal cord injury, the combination of agency and embodiment conferred by a wearable robotic device is a potential source for enhancing functional recovery [58]. Indeed, the capability to trigger and modulate the robotic action via the BCI (agency) and the augmented sense of proprioception achieved via visual and sensory feedback while moving with the exoskeleton (embodiment) could be effective to prevent maladaptive cortical reorganization after the injury. At the same time, they might favor the users’ acceptability of the device and their fulfillment in daily-life activities assisted by the robotic device [58].

The time spent to accomplish the opening/closing motion exceeded the minimum time allowed by the mechatronic constraints of the two devices by less than 4% in all conditions, suggesting an intuitive control of the BCI. In other words, once the robot-assisted hand motion was triggered, subjects were able to sustain the ERD via the EEG with a few interruptions until task completion.

The results of our study suggest that a certain level of embodiment and proprioception conferred by the exoskeleton may lead to more reliable and intuitive control of the hybrid EEG/EOG interface. Indeed, whereas no statistically significant differences in the across-subjects TTI+PreTTI were observed between the synchronous and asynchronous mode for both devices, values were higher when using the external manipulator rather than the exoskeleton. Furthermore, results obtained for this parameter suggest that the control of the exoskeleton exhibits better reliability than the external manipulator control, with 75% of the values remaining below 3 s and below 5 s, for the two devices respectively. In the asynchronous mode, reliability for exoskeleton control further improved, with 75% of trials having a TTI+Pre-TTI below 1 s.

The Pre-TTI and TTI results were analyzed separately to investigate to which extent users were able to anticipate the motor-imagery trigger, and if changes could be observed in the anticipation time between the use of the exoskeleton and the external manipulator. In addition, while the synchronous operation is expected to provide better performances by guiding the user about the timing to activate the triggers, the asynchronous mode would be preferred to increase system flexibility and confer upon users more freedom in performing ADLs [59]. The results for the two control modes were not statistically significant. This could be explained considering that, although no feedback about the timing of EEG activation was given in the asynchronous mode, the possibility to trigger the sub-tasks execution was always supervised by the finite-state machine, which filtered false positives that would have cause improper triggering of the robotic assistance. In addition, in the asynchronous mode, no instructions were given to the users when performing the tasks, so the users could perform the actions as fast as possible or to take their time. This creates a greater sense of control over the system, but on the other hand it has been difficult for us to find differences between the two control modes. With the asynchronous control mode, we intended to verify if the system performance would improve when allowing users to anticipate the closing/opening action of the hand exoskeleton during the reaching movement, without the additional visual feedback to instruct them. However, in the results obtained for the Pre-TTI parameter, we did not observe significant differences between control modes in both assistive devices. Still, results indicate that the anticipation time was greater in the case of the exoskeleton rather than the manipulator. Considering that the BCI and the control mode were the same for both devices, we can hypothesize that the difference observed in the case of the Pre-TTI might be due to a longer anticipation time, which suggests that the system responds faster (and more reliably) to the anticipation of the users with the exoskeleton than with the external robotic device. The best performances in terms of fluency and reliability were achieved for exoskeleton control in asynchronous mode, suggesting the beneficial effect of combining agency (via the active control for the user in asynchronous mode) and embodiment (via the contingent proprioceptive feedback when controlling the exoskeleton) in the use of an assistive device. The higher scores of the NASA-TLX Effort item in the synchronous modality for both devices and of the Mental Demand item for the external manipulator also favor this hypothesis.

We could observe positive effects of the use of the exoskeletons in terms of movement anticipation, which might be explained by the proprioceptive feedback when moving the arm with the exoskeleton. However, some limitations of the study should be discussed. Being a feasibility study, the efficiency of one assistive system over the other could not be demonstrated, because it would have required longer and repeated test sessions. In addition, the study has a relatively small number of patients. Because of this, and because of the diversity of the pathologies of the patients who participated in the study, the results may not be generalizable beyond the conditions of this study. Therefore, this finding should be further explored in clinical studies aimed at verifying the efficacy of such an approach also from a rehabilitation point of view (Additional file 1).

## Conclusions

Although the results of our study in terms of fluency and reliability of EEG control suggest better performances of the exoskeleton over the external manipulator, such results cannot be considered conclusive, due to the heterogeneity of the population under test and the relatively limited number of participants. Furthermore, from the users’ subjective feedback, we could observe a preference toward the control of the external manipulator in terms of lower Physical Demand and Effort and higher perceived Performance (from the NASA-TLX). From the Likert Scale, exoskeleton control was not perceived as sufficiently reliable and practical by 22% of the users. Such results might be due to the highest level of users’ acceptability of external devices, which are intrinsically safer and easier to be used since they are lacking a close physical human-robot interface. The weight (higher than 10 kg) and encumbrance of the whole-arm exoskeleton were limiting factors for the device’s acceptability and participants’ inclination toward its use, stressing the importance of overcoming such technical challenges to effectively translate the use of these devices into people’s daily life.

## Supplementary Information

**Additional file 1.** Detailed parameters per patient.

## Data Availability

The datasets generated during and/or analyzed during the current study are available from the corresponding author on reasonable request.

## Abbreviations

ADLs: Activities of daily living
BCI: Brain–computer interface
BMI: Brain–machine interfaces
DOF: Degrees of freedom
EEG: Electroencephalography
EMG: Electromyography
EOG: Electrooculography
eFE: Elbow flexion-extension
ERD: Event-related desynchronization
FOI: Frequencies of interest
FPGA: Field-programmable-gate-array
FSM: Finite-state machine
HOV: Horizontal eye movements
IMI: Intrinsic motivation inventory
ITI: Inter-trial interval
LRP: Lateralized readiness potential
MEG: Magnetoencephalography
ROM: Range of motion
ROS: Robot operating system
RV: Reference value
sAA: Shoulder adduction–abduction
sFE: Shoulder flexion–extension
sIEr: Shoulder internal–external rotation
SMR: Sensorimotor rhythm
TTI: Times to initialize
PID: Proportional-integrative-derivative
YARP: Yet another robotic platform
