# Supplementary material for: Hybrid brain/neural interface and autonomous vision-guided whole-arm exoskeleton control to perform activities of daily living (ADLs)
Source: J Neuroeng Rehabil. 2023 May 6;20:61. doi: 10.1186/s12984-023-01185-w (PMC10164333; doi:10.1186/s12984-023-01185-w)
Supplement: Supplementary file 1 — Additional file 1. Detailed parameters per patient. [file 12984_2023_1185_MOESM1_ESM.pdf]

Detailed parameters per patient:

						Time to open/close the hand normalized to ERD duration (%)				Pre-TTI (s)				TTI (s)				TTI + Pre-TTI (s)			
						Whole-Arm Exoskeleton		External Robotic Manipulator		Whole-Arm Exoskeleton		External Robotic Manipulator		Whole-Arm Exoskeleton		External Robotic Manipulator		Whole-Arm Exoskeleton		External Robotic Manipulator	
**Patients**	**Sex**	**Age**	**Diagnostic**	**Laterality**	**Barthel Index**	Sync	Async	Sync	Async	Sync	Async	Sync	Async	Sync	Async	Sync	Async	Sync	Async	Sync	Async
1	M	66	Encephalitis	Right	20/20	3,87	1,14	0,51	-	-1,92	-2,09	-1,38	-	6,69	7,29	1,06	-	4,30	3,54	-0,71	-
2	F	62	Stroke	Right	18/20	0,80	1,68	3,74	3,37	-1,37	-1,56	-0,37	-0,81	9,52	7,28	7,35	5,68	6,41	0,65	5,42	2,95
3	M	52	Traumatic Brain Injury Stroke	Right	20/20	1,83	2,80	3,29	4,80	-2,21	-1,70	-1,33	-0,43	4,38	4,99	3,01	6,82	1,34	1,64	1,87	6,16
4	F	20	Spinal Cord Injury C6/7	Right	10/20	2,20	1,71	0,31	0,06	-4,44	-1,64	-0,97	-1,45	4,71	3,07	1,78	1,75	3,05	0,13	0,94	-0,25
5	F	N/A	Traumatic Brain Injury-lived with locked in	Right	16/20	0,55	3,33	1,67	1,26	-2,61	-2,21	-1,13	-0,93	4,40	3,75	5,73	2,43	0,02	0,35	2,59	0,89
6	M	55	Stroke	Right	16/20	0,17	1,52	0,69	1,25	-3,99	-4,11	-0,92	-0,97	5,86	6,17	0,79	3,17	-0,71	-2,18	-0,06	1,39
7	F	N/A	Cerebral Palsy. Ataxia	Right	19/20	0,79	0,73	0,18	0,30	-3,37	-3,12	-0,63	-0,66	1,61	3,06	2,14	1,65	-1,99	-2,50	0,86	0,64
8	M	N/A	Traumatic Brain Injury	Right	19/20	3,63	7,19	1,18	1,33	-1,22	-3,37	-0,85	-0,73	2,41	1,68	7,82	6,29	1,32	-0,85	5,96	4,67
9	M	N/A	Spinal Cord Injury	Right	16/20	0,84	0,40	3,75	3,97	-1,46	-1,70	-1,10	-1,33	4,24	2,68	2,11	6,74	3,10	0,71	1,17	2,71
10	F	56	Stroke	Left	13/20	4,64	-	-	-	-3,47	-	-	-	10,85	-	-	-	3,69	-	-	-

Note. The value shown for each of the parameters corresponds to the mean value of all trials.
